# Topical ropivacaine for analgesia of aplasia cutis congenita in newborns with hereditary epidermolysis bullosa

**DOI:** 10.1186/s13023-020-01605-3

**Published:** 2020-12-01

**Authors:** A. Chambelland, C. Devos, F. Casagrande, C. Chiaverini

**Affiliations:** 1 CRMPR Sud, Department of Dermatology, Université Côte D’Azur, CHU de Nice, Archet 2, 151 route de Saint-Antoine, 06200 Nice, France; 2 Department of Algology, Université Côte D’Azur, CHU de Nice, Nice, France; 3 Department of Neonatal Reanimation, Université Côte D’Azur,CHU de Nice, Nice, France

**Keywords:** Epidemolysis bullosa, Aplasia cutis congenita, Ropivacaine, Pain, Newborn

## Abstract

Aplasia cutis congenita (ACC) in patients with hereditary epidermolysis bullosa (EB) is often associated with major pain. We report our experience with using topical ropivacaine during dressing in newborns with ACC. Eight full-term newborns with EB and ACC were hospitalized in a neonatal intensive care unit for severe pain during dressing despite the use of paracetamol, opioids (n = 8) or ketamine (n = 7). Topical xylocaine was poorly tolerated and not effective. Ropivacaine 2 mg/ml was used directly in contact with the ACC, with a maximum 1 mg/kg/day, which enabled care without the child crying. No immediate or late systemic toxicity was observed. Topical ropivacaine 0.2% appears to be an interesting topical analgesic, with good clinical tolerance and rapid action, in newborns with ACC and EB. These data need to be confirmed in a prospective study including pharmacokinetics evaluations.

**Dear editor,**

Inherited epidermolysis bullosa (EB) is a group a rare genodermatoses characterized by skin and/or mucosal fragility leading to post-traumatic blisters [1]. Depending on the level of cleavage in the skin, four types are described: simplex, junctional, dystrophic and Kindler EB. Aplasia cutis congenita (ACC), defined by a localized absence of skin at birth, can be associated with all types of EB and is usually localized on the legs. Its exact frequency is not known but seems not negligible, up to 17.8% in patients with dystrophic forms [2]. Healing is often long with residual atrophic scar due to the atrophy of subcutaneous tissue. Management of these wounds is difficult because of severe pain during dressing changes in a complex neonatal context.

We report our experience with the use of ropivacaine in 8 full-term children (5 males), mean weight 3038.75 g with EB (4 simplex forms, 2 junctional, 2 dystrophic) and ACC seen in our reference center between 2010 and 2018. All newborns were hospitalized in a neonatal intensive care unit because of severe pain, feeding difficulties and complex dressing of skin wounds. Clinical data are summarized in Table 1. Despite the use of oral paracetamol, opioids (n = 8) or ketamine (n = 7) associated with non-pharmacological interventions, children still had pain during care especially on ACC areas, with inconsolable crying, permanent contraction of the face and removing of the involved limb. Two had apnea. Topical xylocaine (2%) was poorly tolerated and not completely efficient in the first 3 children treated. We then used ropivacaine (2 mg/ml), using a syringe with a predetermined maximum dose of 0.1 mg/kg i.e. 0.25 to 0.3 mg for babies from 2.5 kg to 3.4 kg = 0.1 to 0.15 ml = 2 or 3 drops per dressing directly applied on the ACC surface. Due to the slight viscosity of the product, the liquid quickly recovered the wound. Treated ACC was localized in all cases in lower limbs, with a size ranging from 5 to 15 cm^2^ (Fig. 1). The resulting anesthesia was almost immediate and effective, which enabled continuing the care without the child crying, contracted face and removing limbs. The mean treatment duration was 26.7 days, with 3 dressings per week, corresponding to the time required for wound healing. No immediate allergic, cardiac, digestive or neurologic complication or late neurological systemic toxicity was observed at a median follow-up of 21.5 months (range 12–68).

**Table 1:** Clinical data of patients enrolled in this study

Patient	Age	Birth weight	Term	EB type	ACC	Background therapy	Premedication before care	Ropivacaine 2 mg/ml quantity per day/ duration	Non pharma-cological analgesic	Apnea during care
1	5.5 y	3000 g	41w	AR dystrophic EB, severe	2 feet	Paracetamol, morphine, ketamine, amitriptyline	Morphine, ketamine, NO	0.5 to 1 mg/4 y	Sucrose	–
2	4.5 y	2530 g	38w	Junctional intermediate	2 feet, 2 wrists	Paracetamol, morphine	Morphine, ketamine	0.5 mg/30 d	Sucrose	Yes
3	4.5 y	3400 g	38w	AD EB, simplex severe	left ankle	Paracetamol, amitriptyline	Morphine	0.05 mg/31 d	Sucrose	–
4	20 m	3380 g	38w	AD EB simplex, severe	2 legs	Paracetamol, morphine, ketamine, then sedated condition then paracetamol, morphine, midazolam, amitriptyline, chlorpromazine	Morphine, ketamine, NO then morphine, chlorpromazine	0.2 mg/30 d	Sucrose	Yes
5	13 m	3150 g	40w	AD EB simplex, severe	2 ankles	Paracetamol	Morphine, ketamine, amitriptyline	0.2 mg/45 d	Sucrose	–
6	9 m	3170 g	37w	Junctional intermediate	2 knees, feet, forearms and hands	Paracetamol, morphine	Morphine, ketamine	0.8 mg/14 d	Sucrose	–
7	8 m	2680 g	37w	AD EB simplex, severe	2 legs	Paracetamol, morphine, ketamine,	Morphine, ketamine, midazolam	0.2 mg/10 d	Sucrose	–
8	6 m	3000 g	39w	AR dystrophic EB, severe	2 feet	Paracetamol	Morphine	0.4 mg/10 d	Sucrose	–

*EB* Epidermolysis bullosa, *ACC* aplasia cutis congenita, *AD* autosomal dominant, *AR* autosomal recessive, *NO* nitrous oxide

**Fig. 1:**
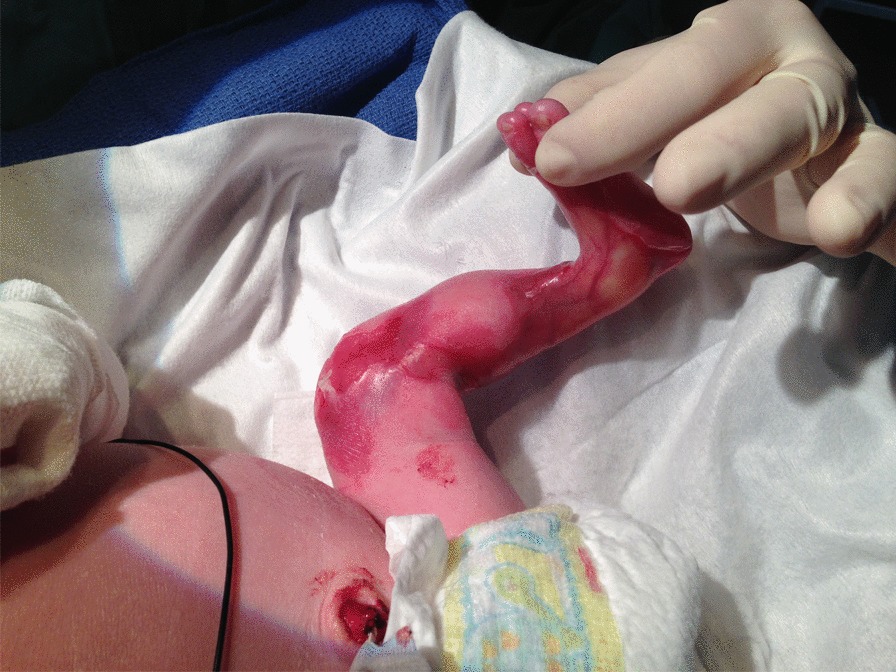
Aplasia cutis congenita of the left leg in a newborn with dominant epidermolysis bullosa simplex, severe

Painful stimuli are stressful for infants, with medium- and long-term consequences for the brain, emotional and behavioral development. The children with ACC we describe still had localized severe pain on ACC areas during dressing despite level-3 analgesics, which led us to find an alternative topical analgesic treatment. Morphine does not provide adequate analgesia for acute procedural pain among preterm neonates [3]. Viscous lidocaine gel 2% leads to rapid analgesia but may exacerbate pain initially. It was not well tolerated nor sufficiently effective in our first patients with ACC. Ropivacaine 0.2%, applied once in direct contact with the ACC, enabled continuing the care with good analgesia without increasing systemic analgesics. Unfortunately, we did not prospectively use a specific pain score for assessing the pain before and after treatment. The DAN scale developed to assess pain during painful procedures in newborns or infants in neonatal cares seems to be relevant. It evaluates three items: facial expression, limb movement and vocal expression [4]. Retrospectively, with available clinical data, all patients had the maximal score (10/10) before the use of ropivacaine with an improvement of at least one point of all parameters after treatment (7/10). Ropivacaine 0.2% is usually employed for regional anesthesia in pediatric patients, including neonates and infants with a dose ranging from 0.25 to 2 mg/kg [5]. Its quick and long-lasting action (> 90 min) is appropriate for use during care of a dressing. Topical application of ropivacaine on mucosae has been described in adults with oral aphtosis [6] and in children after tonsillectomy to reduce postoperative pain [7, 8], from 5 to 10 mg/ml (5 ml/surgery), while the topical use for skin analgesia is not reported. Reported side effects are: allergy, hypo or hypertension, cardiac rhythm anomalies, vomit, convulsion, urine retention, fever, hypothermia, dyspnea. With no available serum assay, we then chose a very low dose (0.1 mg/kg) to limit potential side effects and only in patients monitored in an intensive care unit. No adverse event has been observed. These retrospective findings need to be confirmed in a prospective study including pharmacokinetics evaluations.

In conclusion, topical ropivacaine 0.2% appears to be an interesting skin analgesic with good clinical tolerance and rapid analgesia in newborns with ACC and EB.

## Data Availability

The datasets used and/or analyzed during the current study are available from the corresponding author on reasonable request.
